# Transcriptomic analysis of glycan-processing genes in the dorsal root ganglia of diabetic mice and functional characterization on Ca_v_3.2 channels

**DOI:** 10.1080/19336950.2020.1745406

**Published:** 2020-03-31

**Authors:** Robin N. Stringer, Joanna Lazniewska, Norbert Weiss

**Affiliations:** a Institute of Organic Chemistry and Biochemistry, Czech Academy of Sciences, Prague, Czech Republic; b Third Faculty of Medicine, Charles University, Prague, Czech Republic

**Keywords:** Glycosylation, transcriptome, DRG neurons, diabetes, calcium channel, Ca_v_3.2 channel, T-type channel

## Abstract

Ca_v_3.2 T-type calcium channels play an essential role in the transmission of peripheral nociception in the dorsal root ganglia (DRG) and alteration of Ca_v_3.2 expression is associated with the development of peripheral painful diabetic neuropathy (PDN). Several studies have previously documented the role of glycosylation in the expression and functioning of Ca_v_3.2 and suggested that altered glycosylation of the channel may contribute to the aberrant expression of the channel in diabetic conditions. In this study, we aimed to analyze the expression of glycan-processing genes in DRG neurons from a leptin-deficient genetic mouse model of diabetes (*db*/*db*). Transcriptomic analysis revealed that several glycan-processing genes encoding for glycosyltransferases and sialic acid-modifying enzymes were upregulated in diabetic conditions. Functional analysis of these enzymes on recombinant Ca_v_3.2 revealed an unexpected loss-of-function of the channel. Collectively, our data indicate that diabetes is associated with an alteration of the glycosylation machinery in DRG neurons. However, individual action of these enzymes when tested on recombinant Ca_v_3.2 cannot explain the observed upregulation of T-type channels under diabetic conditions.

**Abbreviations:** Galnt16: Polypeptide N-acetylgalactosaminyltransferase 16; B3gnt8: UDP-GlcNAc:betaGal beta-1,3-N-acetylglucosaminyltransferase 8; B4galt1: Beta-1,4-galactosyltransferase 1; St6gal1: Beta-galactoside alpha-2,6-sialyltransferase 1; Neu3: Sialidase-3

## Introduction

It is well established that increased expression of the low-voltage-activated Ca_v_3.2 T-type calcium channel within neurons of the dorsal root ganglia contribute to the sensitization of nociceptive sensory fibers in response to hyperglycemia associated with diabetes, leading to painful symptoms of peripheral diabetic neuropathy [1–3]. This notion is further exemplified by the observation that pharmacological blockade of T-type channels alleviates diabetes-induced hyperalgesia in a leptin-deficient genetic mouse model of diabetes (*ob*/*ob*) [4]. Furthermore, it has been reported that removal of terminal sialic acid moieties from complex glycan structures can normalize T-type currents in DRG neurons isolated from *ob*/*ob* mice, and reverse neuropathic pain *in vivo* [5], suggesting that glycosylation of Ca_v_3.2 could possibly represent an underlying mechanisms contributing to the enhanced expression of the channel during diabetes.

Protein glycosylation is a posttranslational modification that refers to the co-valent addition of a sugar molecule oligosaccharide (glycan) to specific residues within the target protein. It is an essential chemical process that contributes to the proper maturation, sorting, and functioning of proteins including ion channels [6,7], and several studies have documented the importance of glycosylation for the expression of Ca_v_3.2 channels [8,9]. However, the underlying cellular mechanisms by which Ca_v_3.2 channel may undergo aberrant glycosylation during diabetes have remained unknown.

In this study, we aimed to specifically analyzed the transcriptomic profile of glycan-modifying enzymes in DRG neurons from diabetic *db*/*db* mice and assess the effect of these enzymes on the expression of recombinant Ca_v_3.2 channels.

## Materials and methods

### Animals

8 weeks old male *db*/*db* mice and their control hibernates were purchased from Janvier Labs and were kept under standard conditions for 3 weeks to allow sufficient adaptation. The mean glycemia values measured using a glucocard X-meter ARKAY from blood samples drawn from the tail were 8.2 ± 0.6 mmol/L for wild-type animals (*n* = 6) and 29.9 ± 0.7 mmol/L for *db*/*db* animals (*n* = 7).

### Transcriptomic analysis

Transcriptomic analysis of glycan-modifying enzymes was performed on total RNA harvested from the dorsal root ganglia (lumbar L4/L6) of wild-type and *db*/*db* mice using the Glycosylation RT2 Profiler PCR Array (Qiagen) according to the manufacturer’s instructions. The PCR array and qRT-PCR were performed on a LightCycler® 480 (Roche) with the following PCR conditions: 95°C for 5 min, 40 cycles at 95°C for 15 sec, 60°C for 15 sec, and 72°C for 20 sec. Each test was run three times and the mean values were taken to eradicate any discrepancies. 84 key genes encoding glycan-processing enzymes were analyzed and included glycosyltransferase and glycosidase for several important sugars (galactose, glucose, mannose, N-acetylgalactosamine, N-acetylglucosamine, fucose and sialic acid).

### Plasmid cDNA constructs

The cDNA construct encoding for the human Ca_v_3.2 wild-type in pcDNA3.1 was previously described [10]. The plamid cDNAs encoding for the human glycan-modifying enzymes Galnt16, B3gnt8, B4galt1, St6gal1, and Neu3 in pCMV3 were purchased from Sino Biological.

### Cell culture and heterologous expression

Human embryonic kidney tsA-201 cells were grown in high glucose DMEM medium supplemented with 10% fetal bovine serum and 1% penicillin/streptomycin (all media were purchased from Invitrogen) and maintained under standard conditions. Cells were transfected using the calcium/phosphate method using 2.5 ± g of Ca_v_3.2 plasmid and 2.5 ± g of plasmid encoding for the glycan-modifying enzymes. For transfections using the channel alone, 2.5 ± g of empty pcDNA3 vector was added to the mixture to maintained the total amount of cDNA.

### Electrophysiology

Patch clamp recording of T-type currents in tsA-201 cells expressing Ca_v_3.2 channels was performed 72 h after transfection in the whole-cell configuration at room temperature (2224°C) as previously described [11]. The external solution contained (in millimolar): 5 BaCl_2_, 5 KCl, 1 MgCl_2_, 128 NaCl, 10 TEA-Cl, 10 D-glucose, 10 4-(2-hydroxyethyl)-1-piperazineethanesulfonic acid (HEPES) (pH 7.2 with NaOH). Patch pipettes were filled with an internal solution containing (in millimolar): 110 CsCl, 3 Mg-ATP, 0.5 Na-GTP, 2.5 MgCl_2_, 5 D-glucose, 10 EGTA, and 10 HEPES (pH 7.4 with CsOH), and had a resistance of 2–4 MΩ. Recordings were performed using an Axopatch 200B amplifier (Axon Instruments) and acquisition and analysis were performed using pClamp 10 and Clampfit 10 software, respectively (Axon Instruments). The linear leak component of the current was corrected online and current traces were digitized at 10 kHz and filtered at 2 kHz. The voltage dependence of activation of Ca_v_3.2 channels was determined by measuring the peak T-type current amplitude in response to 150 ms depolarizing steps to various potentials applied every 10 s from a holding membrane potential of −100 mV. The current-voltage relationship (I/V) curve was fitted with the following modified Boltzmann Equation (1):

(1) $$I\left(V\right)=Gmax\frac{\left(V-Vrev\right)}{1+exp\frac{\left(V0.5-V\right)}{k}}$$

with *I*(*V*) being the peak current amplitude at the command potential *V, G*_max_ the maximum conductance, *V*_rev_ the reversal potential, *V*_0.5_ the half-activation potential, and *k* the slope factor. The voltage dependence of the whole-cell Ba^2+^ conductance was fitted with the following modified Boltzmann Equation (2):

(2) $$G\left(V\right)=\frac{Gmax}{1+exp\frac{(\left(V0.5-V\right)}{k}}$$

with *G*(*V*) being the Ba^2+^ conductance at the command potential *V*.

The voltage dependence of the steady-state inactivation of Ca_v_3.2 channels was ascertained by measuring the peak T-type current amplitude in response to a 150 ms depolarizing step to −20 mV applied after a 5 s-long conditioning prepulse ranging from −120 mV to −30 mV. The current amplitude obtained during each test pulse was normalized to the maximal current amplitude and plotted as a function of the prepulse potential. The voltage dependence of the steady-state inactivation was fitted with the following two-state Boltzmann function (3):

(3) $$I\left(V\right)=\frac{Imax}{1+exp\frac{\left(V-V0.5\right)}{k}}$$

with *I*_max_ as the maximal peak current amplitude and *V*_0.5_ as half-inactivation voltage.

The recovery from inactivation was determined using a double-pulse protocol from a holding potential of −100 mV. The cell membrane was depolarized for 2 s at 0 mV (inactivating prepulse) to ensure complete inactivation of the channel, and then to −20 mV for 150 ms (test pulse) after an increasing time period (interpulse) ranging between 0.1 ms and 2 s at −100 mV. The peak current from the test pulse was plotted as a ratio of the maximum prepulse current versus interpulse interval. The data were fitted with the following single-exponential function (4):

(4) $$\frac{I}{Imax}=A\times \left(1-exp\frac{-t}{\tau }\right)$$

where τ denotes the time constant of channel recovery from inactivation.

### Statistical analysis

Data values are presented as mean ± S.E.M. for *n* measurements. Statistical analysis was performed using GraphPad Prism 7. Statistical significance was determined using a one-way ANOVA test and datasets were considered significantly different for *p* ≤ 0.05.

## Results

### Expression of glycan-processing enzymes in the dorsal root ganglia of diabetic mice

**Figure 1.**
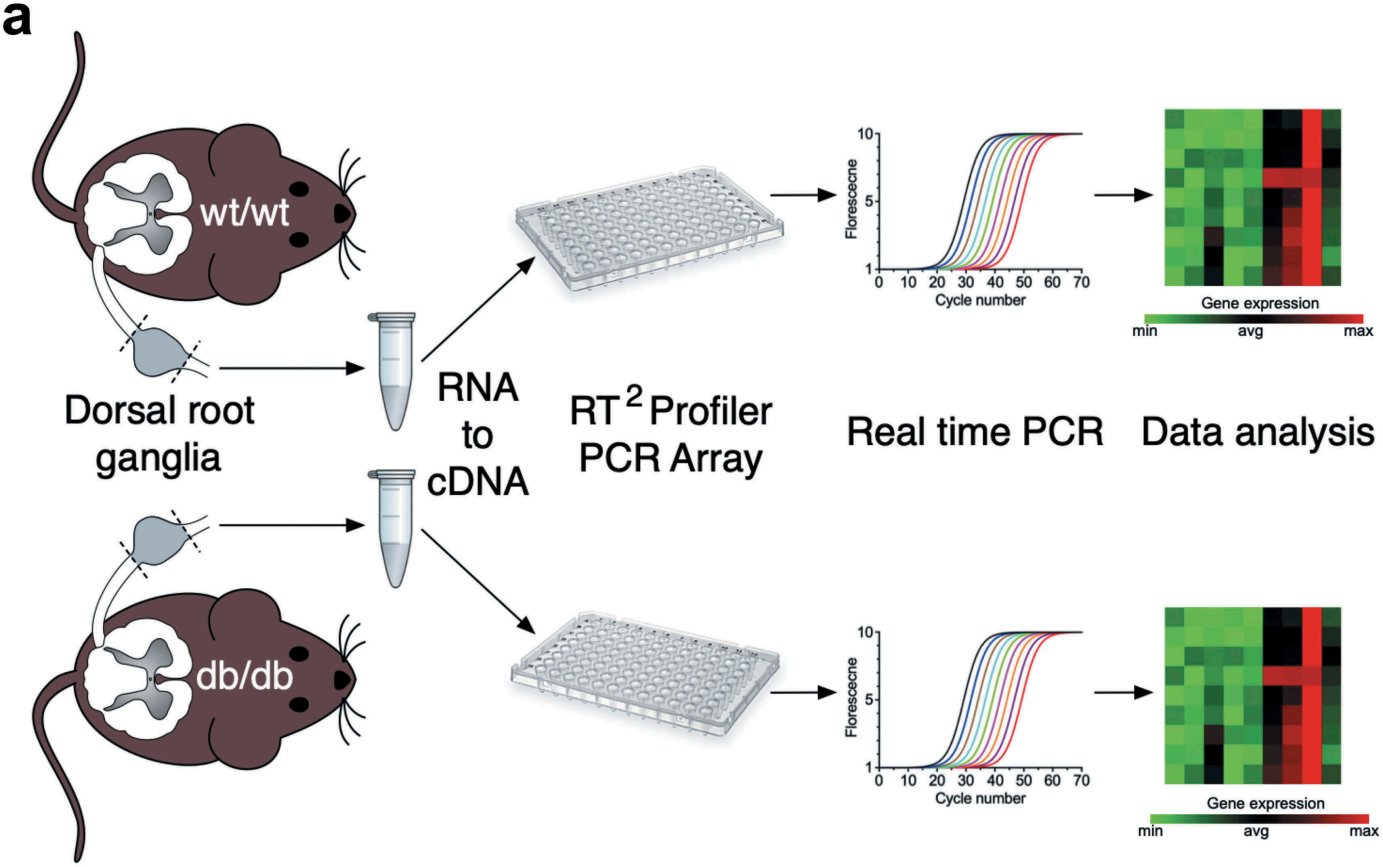
Schematic representation of the transcriptomic analysis process. (a) Total RNA harvested from the dorsal root ganglia (lumbar L4/L6) of wild-type and *db*/*db* mice and subjected to the Glycosylation RT^2^ Profiler PCR Array to analyze the expression level of 84 genes encoding for glycan-processing enzymes.

**Figure 2.**
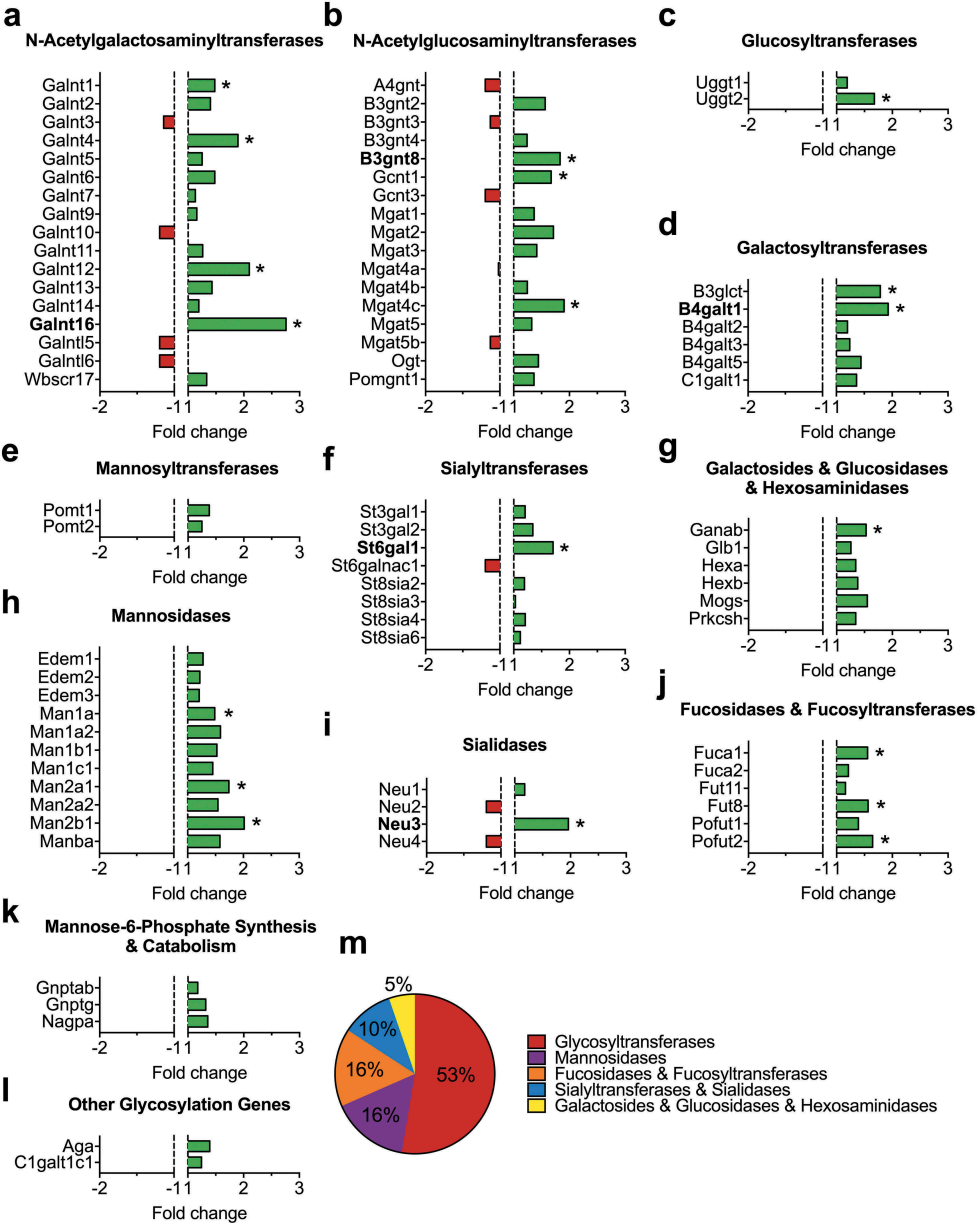
Summary of the transcriptomic profiling of glycan-modifying genes in the dorsal root ganglia of *db*/*db* mice. Data are presented as fold change compared to wild-type animals for (a) N-acetylgalactosaminyltransferases, (b) N-acetylglucosaminyltransferases, (c) Glucosyltransferases, (d) Galactosyltransferases, (e) Mannosyltransferases, (f) Sialyltransferases, (g) Galactosides/Glucosidases/Hexosaminidases, (h) Mannosidases, (i) Sialidases, (j) Fucosidases/Fucosyltransferases, (k) Mannose-6-Phosphate synthesis/catabolism, and (l) other glycosylation genes. Enzymes indicated in bold were functionally characterized on Ca_v_3.2 channels. (m) Summary of up-regulated genes.

In order to assess the expression of glycan-processing enzymes in diabetic conditions, we performed a differential transcriptomic analysis on the dorsal root ganglia isolated from a transgenic mouse model of diabetes (*db*/*db*) versus wild-type animals (Figure 1). 19 out of 84 enzymes analyzed were found significantly upregulated (*p* < 0.05) in diabetic conditions (Figure 2am). The majority of these enzymes (53%) belonged to the family of glycosyltransferases (Galnt1, Galnt4, Galnt12, Galnt16, B3gnt8, Gcnt1, Mgat4 c, Uggt2, B3glct, and B4galt1) that catalyze the transfer of saccharide moieties from an activated nucleotide sugar to a nucleophilic glycosyl acceptor molecule. In addition, 16% belonged to the family of mannosidases (Man1a, Man2a1, and Man2b1) that hydrolyze mannose moieties; 16% to the family of fucosidases/fucosyltransferases (Fuca1, Fut8, and Pofut2); 5% to the family of galactosides/glucosidases/hexosaminidases (Ganab); and 10% to the family of sialidases/sialyltransferases (St6gal1, and Neu3) involved in the processing of sialic acid moieties from complex glycan structures (Figure 2m). In contrast, we did not observe any enzymes that were significantly down-regulated.

### Functional effect of glycan-processing enzymes on the expression of recombinant Ca_v_3.2 channels

**Figure 3.**
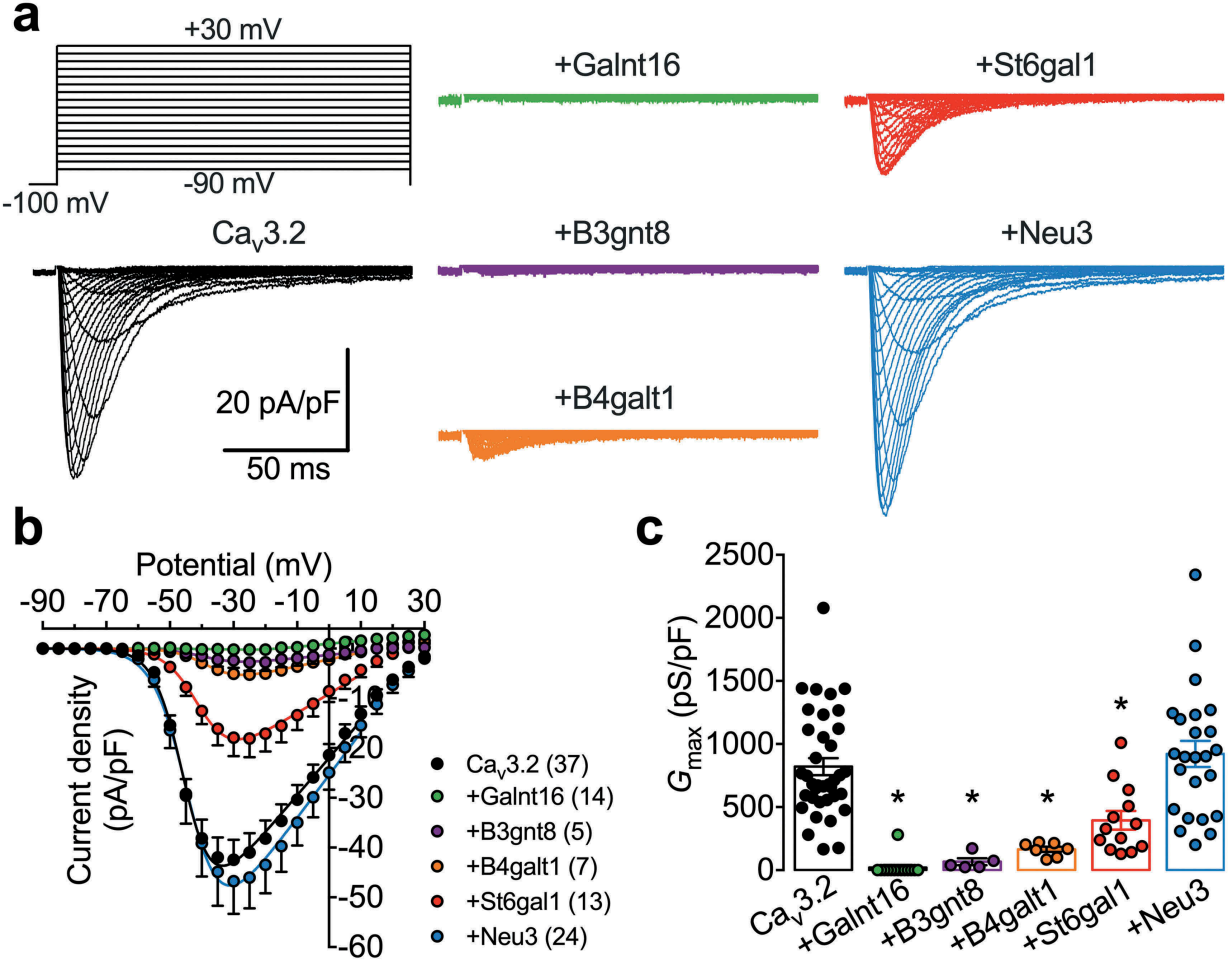
Influence of glycan-modifying enzymes on the functional expression of Ca_v_3.2 channels. (a) Representative T-type current traces recorded from cells expressing Ca_v_3.2 alone (black traces) and in combination with Galnt16 (green traces), B3gnt8 (purple traces), B4galt1 (orange traces), St6gal1 (red traces), Neu3 (blue traces) glycan-modifying enzymes in response to 150 ms depolarizing steps ranging between −90 mV and 30 mV from a holding potential of −100 mV. (b) Corresponding mean peak current density-voltage (*I*/*V*) relationship. (c) Corresponding mean maximal macroscopic conductance (*G*_max_) values obtained from the fit of the *I*/*V* curves with the modified Boltzmann equation (1).

Next, we aimed to assess the functional impact of up-regulated glycan-modifying enzymes on recombinant Ca_v_3.2 channels expressed in tsA-201 cells. Six enzymes responsible for the processing of the glycan structure at different stages were assessed: Galnt16 (*N*-acetylgalactosaminyltransferase) responsible for catalyzing the initial addition of N-acetylgalactosamine to a serine or threonine residue on early protein precursors [12]; B3gnt8 (*N*-acetylglucosaminyltransferase) responsible for the elongation of the polylactosamine chains on tetraantennary *N*-glycans [13]; B4galt1 (Galactosyltransferase) which catalyzes the addition of galactose moieties to N-acetylglucosamine of complex *N*-glycans in the Golgi apparatus [14]; St6gal1 (Sialyltransferase) responsible for catalyzing the final transfer of sialic acid moieties from CMP-sialic acid to galactose acceptor substrates [15]; and Neu3 (sialidase) expressed in the plasma membrane and responsible for removing sialic acid moieties from glycoproteins and glycolipids, acting in the opposite way of St6gal1 [16]. Representative T-type current traces recorded from cells co-expressing Ca_v_3.2 with glycosyltransferases (Galnt16, B3gnt8, or B4galt1) and sialic acid-modifying enzymes (St6gal1 or Neu3) are shown in Figure 3a in response to 150 ms depolarizing steps ranging between −90 mV and 30 mV from a holding potential of −100 mV. Unexpectedly, co-expression of glycosyltransferases with Ca_v_3.2 nearly abolished T-type currents. For instance, the maximal T-type conductance (*G*_max_) in cells expressing Ca_v_3.2 with Galnt16, B3gnt8, and B4galt1 was reduced by 98% (*p* = 0.0001) (20 ± 20 pS/pF, *n* = 14), 92% (*p* = 0.0003) (67 ± 28 pS/pF, *n* = 5), and 80% (*p* = 0.0002) (165 ± 21 pS/pF, *n* = 7), respectively, compared to cells expressing Ca_v_3.2 alone (821 ± 68 pS/pF, *n* = 37) (Figure 3b,c). We also observed a significant decrease of *G*_max_ in cells co-expressing the sialyltransferase St6gal1 by 52% (*p* = 0.0028) (395 ± 74 pS/pF, *n* = 13) (Figure 3ac and Table 1). In contrast, we did not observe a significant alteration (*p* = 0.7542) of *G*_max_ in cells co-expressing the sialidase Neu3 (921 ± 104 pS/pF, *n* = 24) (Figure 3ac and Table 1). Altogether, these data indicate that some of the glycan-processing enzymes tested here can have a potent influence on the expression of Ca_v_3.2 that is consistent with a loss-of-channel function.

**Table 1.** Electrophysiological properties of human Ca_v_3.2 channels expressed in tsA-201 cells in the presence of sialic acid-processing enzymes.

	Activation					Inactivation			RFI	
Channel	V_0.5_ (mV)	*k*	(n)	*G*_max_ (pS/pF)	(n)	V_0.5_ (mV)	*k*	(n)	τ (ms)	(n)
Ca_v_3.2	−43.6 ± 0.6	4.5 ± 0.2	37	820 ± 68	37	−68.3 ± 0.9	2.9 ± 0.7	13	447 ± 34	11
+St6gal1	−38.7 ± 0.7*	5.5 ± 0.3	13	395 ± 74*	13	−70.7 ± 1.6	3.2 ± 0.3	8	550 ± 48	8
+Neu3	−41.8 ± 0.9	5.3 ± 0.2*	24	921 ± 104	24	−66.6 ± 1.3	3.7 ± 0.2	16	422 ± 39	7

### Electrophysiological properties of Ca_v_3.2 channels in the presence of sialic acid-processing enzymes

**Figure 4.**
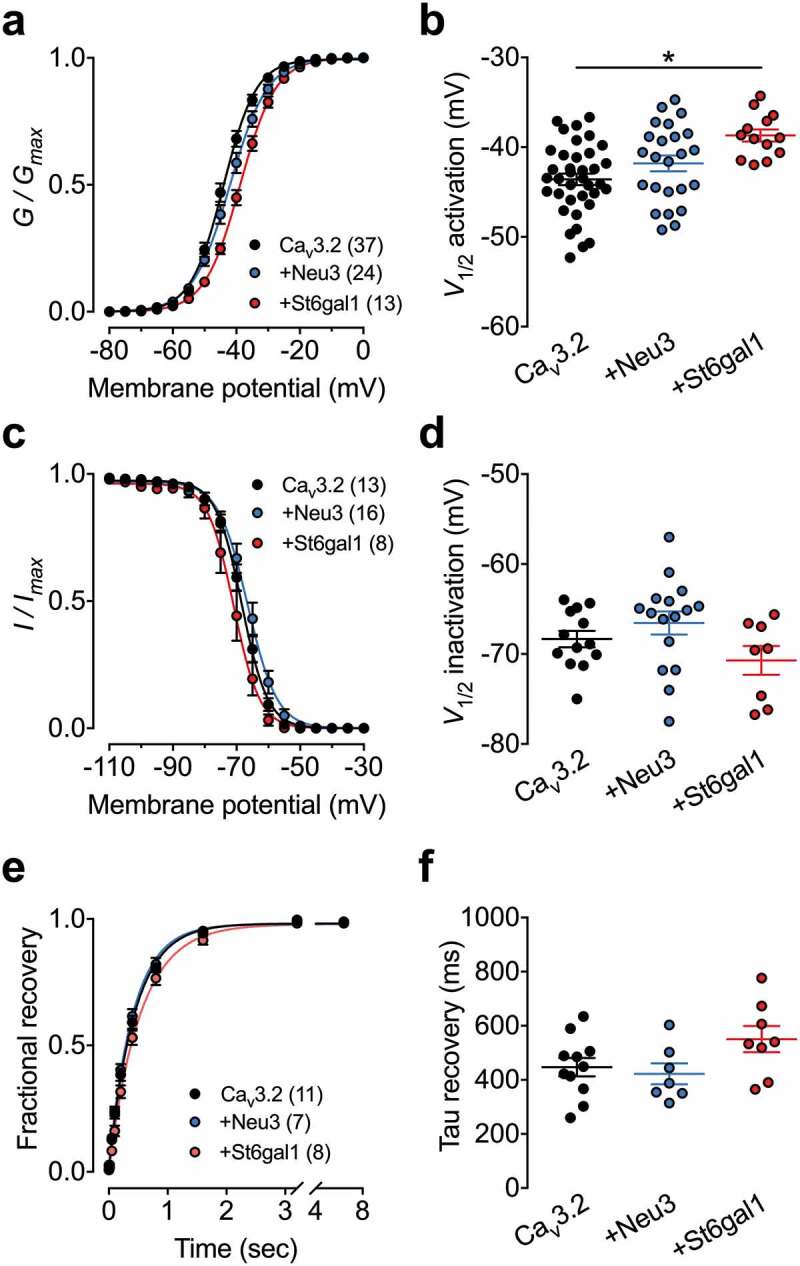
Influence of sialic acid-processing enzymes on the electrophysiological properties of Ca_v_3.2 channels. (a) Mean normalized voltage-dependence of T-type current activation for cells expressing Ca_v_3.2 alone (black circles) and in combination with Neu3 (blue circles) or St6gal1 (red circles). (b) Corresponding mean half-activation potential values obtained from the fit of the activation curves with the modified Boltzmann Equation (2). (c,sd) Legend same as for (a,b) but for the voltage-dependence of steady state inactivation. Half-inactivation potential values was obtained from the fit of the inactivation curves with the two-state Boltzmann function (3). (e) Mean normalized recovery from inactivation kinetics. (f) Corresponding mean time constant values of recovery from inactivation obtained from the fit of the recovery curves with the single-exponential function (4).

Previous studies have shown that the terminal sialic acid moiety attached to complex glycan structures can affect the gating of voltage-gated ion channels [17]. Therefore, we further assessed the voltage-dependence of activation and inactivation of Ca_v_3.2 channels in the presence of the sialyltransferase St6gal1 and sialidase Neu3. The mean half-activation potential in cells expressing St6gal1 was shifted by 4.9 mV (*p* = 0.0001) toward depolarized potentials (−38.7 ± 0.7 mV, *n* = 13) compared to cells expressing the channel alone (−43.6 ± 0.6 mV, *n* = 37) (Figure 4a,b and Table 1). In contrast, co-expression of Neu3 had no significant effect of the voltage-dependence of activation of Ca_v_3.2. Furthermore, neither St6gal1 nor Neu3 altered the voltage-dependence of inactivation (Figure 4c,d and Table 1) or the recovery from inactivation of Ca_v_3.2 channels (Figure 4e,f and Table 1). Altogether, these data indicate that increased sialylation activity tends to negatively modulate recombinant Ca_v_3.2 channels when expressed in tsA-201 cells.

## Discussion

Increased expression of Ca_v_3.2 in primary afferent nociceptive fibers is causally linked to the development of peripheral painful neuropathy associated with nerve injury [18–20], antineoplastic drugs [21–23], inflammation [24,25], and diabetes [1,3]. Several studies have unraveled some of the mechanisms underlying the pathological expression of Ca_v_3.2 and alteration of the posttranslational regulation of the channel including ubiquitinylation [26], SUMOylation [27] and phosphorylation [28,29]. Defects in these processes have emerged as some of the primary reasons leading to enhanced expression of the channel. Furthermore, altered glycosylation of Ca_v_3.2 was proposed to contribute to the sensitization of nociceptive fibers in response to hyperglycemia associated with diabetes [5]. In this study, we show using a differential transcriptomic approach that several glycan-modifying enzymes are upregulated in DRG neurons from *db*/*db* mice compared to wild-type animals. These results are consistent with previous studies reporting an alteration of glycan-processing enzymes in the kidney of diabetic mice [30]. Several of these enzymes contribute to the processing of important sugars including glucose, galactose, mannose, and fucose, and therefore alteration of their expression level could potentially alter the processing and maturation of the glycan structures. Furthermore, we found that several enzymes involved in the processing of the terminal sialic acid moieties found in complex glycan structures were upregulated in diabetic conditions. This aspect is particularly relevant in the context of PDN since enzymatic removal of sialic acid moieties with neuraminidase was reported to normalize T-type currents in DRG neurons isolated from diabatic mice and to alleviate PDN *in vivo* [5]. Furthermore, sialylation was reported to contribute to the hyperexcitability of DRG neurons following peripheral nerve injury [31]. However, our functional analysis on recombinant Cav3.2 channels did not provide evidence in support of a role for these enzymes in the upregulation of Ca_v_3.2 when co-expressed individually with the channel. For instance, co-expression of glycosyltransferases Galnt16, B3gnt8, and B4galt1 with Ca_v_3.2 produced an almost complete loss of functional expression of the channel. However, several studies have previously shown that glycosyltransferases can form heterodimers that contribute to their subcellular expression, enzymatic activity, efficient biosynthesis of glycan chains, trafficking through intracellular vesicles, and substrate specificities [32]. For instance, binding of B3gnt8 appears to cause a conformational change in the catalytic site of B3gnt2 and increases its enzymatic activity [33]. Therefore, we cannot exclude that overexpression of individual enzymes with Ca_v_3.2 in tsA-201 cells as performed in our study may not fully capture the more complex situation in DRG neurons where the expression several genes encoding for glycan-modifying enzymes is altered at the same time and there could be synergetic effects among the various players. Furthermore, tsA-201 cells were grown in high glucose medium which represents another variable that could have influenced the phenotypic effect of these enzymes on Ca_v_3.2 channels. In contrast to glycosyltransferases, co-expression of the sialyltransferase St6gal1 produced a relatively mild decreased expression of the channel with a depolarized shift of the voltage-dependence of activation, indicating that sialylation contributes to the functioning of Ca_v_3.2. However, co-expression of the neuraminidase Neu3 that removes sialic acid moieties did not altered expression of the channel, nor its gating properties. These results are consistent with previous studies showing that application of neuraminidase on tsA-201 cells expressing Ca_v_3.2 channels did not alter channel function [8], which could suggest a low basal level of sialylation in these cells.

Altogether, this study identified several glycan-modifying genes whose expression is altered in DRG neurons under diabetic condition. However, we did not find evidence for a role of these enzyme in the up-regulation of Ca_v_3.2 channels. At this stage, we cannot exclude that expression of glycan-modifying enzymes in DRG neurons may have produced a different phenotypic effect on Ca_v_3.2 and this aspect would deserve further investigations in native conditions.
